# Boosting Formic Acid Production in Mildly Acidic Media—The Role of Native Surface Oxide on the CO_2_ Reduction Performance

**DOI:** 10.1021/acsomega.5c10136

**Published:** 2025-11-11

**Authors:** Thomas Mairegger, Christoph Griesser, Sergio Díaz-Coello, Michael Höltig, Christoph Gimmler, Philipp Stadler, Alexander Beck, Julia Kunze-Liebhäuser

**Affiliations:** † Department of Physical Chemistry, 27255 University of Innsbruck, Innrain 52c, 6020 Innsbruck, Austria; ‡ Net Zero Emission Labs GmbH, Sinning 1, 83101 Rohrdorf, Germany; § Fraunhofer IAP-CAN, Grindelallee 117, 20146 Hamburg, Germany

## Abstract

Bismuth catalysts hold significant promise for the electroreduction of CO_2_ to formic acid (FA), yet their performance in acidic electrolytes remains largely unexplored despite the clear advantages for product separation and direct industrial integration. Here, we systematically investigate the catalytic activity and long-term stability of bismuth catalysts under acidic conditions (pH 3). At this pH, the hydrogen evolution reaction bifurcates into distinct proton and water reduction pathways with the CO_2_ reduction reaction to FA taking place exactly between them. We demonstrate that bismuth catalysts efficiently suppress the proton reduction, leading to over 95% selectivity toward FA in acidic media. Furthermore, long-term measurements reveal the governing catalytic role of an inherently formed surface oxide, which develops upon catalyst ink preparation in ambient air, even if metallic bismuth is used as catalyst. This is evidenced by cyclic voltammetry, X-ray photoelectron spectroscopy, and potentiostatic product detection via online gas chromatography. Our work reports on successful electrocatalytic formic acid production from CO_2_ at pH 3, which opens viable pathways for the implementation of this reaction through proton-exchange membrane technologies.

## Graphical abstract

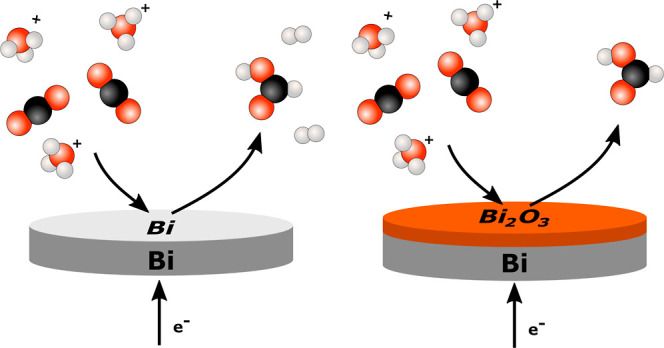

## 1 Introduction

The increasing concentration of atmospheric CO_2_ poses a severe global challenge,^1^ necessitating innovative strategies for carbon capture and utilization. Among the various approaches, the electrochemical CO_2_ reduction reaction (CO_2_RR) has emerged as a promising pathway. This process can sustainably convert CO_2_ into valuable chemicals and fuels using renewable electricity, thereby tackling environmental concerns and promoting a circular carbon economy.

While the CO_2_RR can yield diverse hydrocarbons and oxygenates, the initial industrial focus is on products containing only one carbon atom, such as formic acid (FA) and carbon monoxide (CO). These are favored due to the high Faraday efficiencies (FEs) and partial current densities during their formation, achievable with existing catalysts, which simplifies product separation and increases process viability.^2,3^ Presently, formic acid (FA) and carbon monoxide (CO) are primarily synthesized from fossil fuels through multistep processes.^1,4^ For instance, the conventional production of formic acid typically involves CO carbonylation, followed by a sequence of hydrolysis, distillation, extraction, and dehydration.^1^ In stark contrast, the electrochemical reduction of CO_2_ offers a compelling sustainable alternative, enabling the direct, single-step conversion of CO_2_ to FA. Beyond its traditional uses in agriculture, textiles, and leather,^5^ FA shows promise as a hydrogen carrier,^6,7^ a key biotechnological building block,^8,9^ and a cement curing accelerator^10^. This last application is particularly interesting for cement plant circularity,^11,12^ given their significant annual CO_2_ emissions (7–8% of the worldwide CO_2_ emissions) resulting from inherent processes (calcination step: CaCO_3_ → CaO + CO_2_) and the high temperatures (>1500 °C) required in the rotary kiln.

Within this context, the selective production of FA in acidic media warrants particular attention. While much CO_2_RR research focuses on alkaline conditions, which is often favored for suppressing the competing hydrogen evolution reaction (HER), acidic environments offer unique benefits for FA production.^13–15^ These include simplified downstream separation of liquid FA^16^ and mitigation of carbonate formation and precipitation, common issues in alkaline or neutral electrolytes, which can deactivate catalysts and clog membranes.^13,17^ Further, industrial applications demand exceptional membrane stability, with operational lifetimes of several thousand hours being crucial for commercial viability.^18^ Here, the acidic operating environment inherent to proton exchange membrane (PEM) systems offers a distinct advantage. PEMs currently demonstrate superior durability, often exceeding 20,000 h of operation, significantly surpassing the typical ∼2000 h lifespan observed for anion exchange membranes (AEMs).^19^ Therefore, understanding the catalytic pathways for FA production in acidic environments, especially with the aim of effective HER suppression, is crucial for the development of viable CO_2_RR technology development. In this regard, bismuth (Bi)-based catalysts are compelling due to their high activity and selectivity toward FA production and their high overpotential for the competing HER.^20–25^ However, despite these advantages, the practical application of Bi-based catalysts faces considerable hurdles. Their long-term stability and sustained selectivity, particularly when operating under highly acidic conditions, remain a significant challenge.^14,22^ These harsh environments can induce catalyst dissolution, surface restructuring, or passivation, compromising their durability and consistent performance over extended operational periods.^14,22^ Considering these inherent limitations, careful control of the electrolyte pH is a crucial optimization strategy. Maintaining a pH of approximately 3 emerges as the most promising operational window. This moderately acidic condition enables the simultaneous promotion of FA formation, aligning favorably with its p*K*_a_ of 3.77, to ensure the formation of the desired protonated product. Concurrently, it critically contributes to the mitigation of the undesirable HER, a beneficial effect directly attributed to the reduced proton concentration in the solution.^13^ However, a critical practical challenge arises because FA is the main product, causing the solution to gradually acidify and making the maintenance of pH 3 difficult over time. Therefore, the effect of this gradual change in bulk pH on the overall CO_2_RR performance warrants further investigation.

This study investigates the catalytic performance of a carbon-supported nanoporous Bi catalyst in a mildly acidic environment. We focused on an acidified K_2_SO_4_ electrolyte (pH 3), which has been reported to be conducive for efficient CO_2_ reduction.^26–29^ At this pH, the HER has two distinct reduction pathways: diffusion-limited proton reduction and water reduction.^30,31^ Notably, the CO_2_RR is situated exactly between the onset potentials of the two HER pathways. Our study reveals the crucial role of the native surface oxide in dictating the catalytic performance for CO_2_ reduction in acidic media, which inherently develops during ink preparation in ambient air. While the influence of oxides has been extensively investigated in neutral and alkaline systems,^23,32–35^ our study highlights its significance in acidic media.

## 2 Experimental Section

### 2.1 Synthesis of Nanoporous Carbon-Supported Bismuth (Bi)

Bi-based nanoparticles have been synthesized via a modification of the procedure reported by Xia et al.^36^ Briefly, 0.2 mmol of ammonium bismuth citrate (99%, Sigma-Aldrich) was dissolved in 10 mL of ultrapure water, and then 1.0 mL of soluble starch solution (10 g/L, 99%, Sigma-Aldrich) was added to the above solution at room temperature under stirring. After 20 min, a 1 M NaBH_4_ (98%, Sigma-Aldrich) aqueous solution was added dropwise to obtain a black dispersion containing the Bi-based nanoparticles. These were purified by centrifuging and rinsing with ultrapure water and washing with a mixture of ethanol (absolute, Sigma-Aldrich) and iso-propanol (>99.8%, Sigma-Aldrich) for several times. X-ray Diffraction (XRD) measurements (Figure S1a) confirm the metallic nature of the Bi nanoparticles, while transmission electron microscopy (TEM; see Figure S1b) reveals a particle size distribution of 3.1 ± 0.8 nm. The pure Bi nanoparticles were not stable at room temperature for an increased period and hence were supported on Carbon Black (Vulcan XC 72, Cabot), yielding a 50/50 wt % ratio of Bi and carbon, which will be denoted as Bi/C throughout this publication.

### 2.2 Preparation of Bi/C Ink and Bi_2_O_3_ Ink

To prepare the Bi/C for electrochemical measurements, 10 mg of Bi/C was mixed with 21 μL of Nafion ionomer (D2021 Nafion solution, Ion Power) in 2 mL of a 2:1 isopropanol/water solution. Sonicating this mixture for 30 min produced a 70/30 wt % Bi/C/Nafion ink containing 0.005 mg/μL of catalyst. For all measurements in this work, 20 μL of this ink was drop-casted onto a 7 mm glassy carbon electrode, achieving a catalyst loading of 0.26 mg/cm^2^.

For comparison, commercial bismuth oxide (Bi_2_O_3_) nanopowders (90–210 nm size, 99.8% pure, Sigma-Aldrich) were prepared under the same conditions as the Bi/C catalyst, yielding an equivalent 0.26 mg of Bi_2_O_3_/cm^2^ loading on the glassy carbon support. Functioning as a reference for discerning the influence of surface oxide, the stable Bi_2_O_3_ nanoparticles were not supported on Carbon Black.

### 2.3 Physicochemical Characterization

XRD studies were performed on a Philips X’Pert PRO MPD diffractometer. The liquid sample was prepared by transferring it to a silicon wafer and allowing it to dry. The resulting XRD pattern (Figure S1a) confirms the successful synthesis of pure Bi nanoparticles with no evidence of Bi_2_O_3_ formation.

TEM studies were performed using a JEOL JEM-1011 microscope. Samples were prepared by depositing the nanoparticle dispersion onto standard TEM grids and allowing them to dry. TEM images (Figure S1b), acquired at an accelerating voltage of 20 kV, revealed that the nanoparticles exhibit a particle size of approximately 3.1 ± 0.8 nm.

X-ray photoelectron spectroscopy (XPS) experiments were conducted on a MultiLab 2000 (Thermo Fisher Scientific) instrument equipped with a monochromatized Al Kα X-ray source (*E* = 1486.6 eV) and a hemispherical sector analyzer. High-resolution spectra were recorded for the Bi 4f region with a step size of 0.1 eV, a dwell time of 0.1 s, and a pass energy of 20 eV. A flood gun was used for charge compensation via emission of electrons at a kinetic energy of 6 eV. Spectra were processed utilizing CasaXPS,^37^ always applying a Shirley-type background and mixed Gaussian–Lorentzian functions (GL30) for deconvolution and fitting.

### 2.4 Electrochemical Characterization

Cyclo voltammograms (CVs) were carried out at room temperature in a classical three-electrode setup, utilizing a SP-150e potentiostat (BioLogic). The all-glass three-electrode cell comprised a Bi/C or Bi_2_O_3_ working electrode (WE), a silver/silver chloride (Ag/AgCl) reference electrode (RE), and a carbon rod counter electrode (CE). The electrolyte was 0.1 M K_2_SO_4_, adjusted to pH 3 by using H_2_SO_4_. The electrolyte pH remains constant at pH 3 even after the solution was purged with CO_2_ for an hour. All reported potentials in this work are referred to the reversible hydrogen electrode (RHE), which is calculated by *V*_RHE_ = *V*_Ag/AgCl_ + 0.199 V + 0.177 V.

### 2.5 Gas Chromatography

The gaseous products were analyzed by using a gas chromatograph (GC; NEXIS GC-2030, Shimadzu), composed of a ShinCarbon ST 100/20 column (2 m, 1 mm ID, 1/16 OD, Silco) for the separation and a thermal conductivity detector (TCD) and a flame ionization detector (FID) for the quantification of the gases. Potentiostatic experiments, coupled with GC, were conducted in an H-cell setup, separated into an anodic compartment and a cathodic compartment by a Nafion 115 membrane (Fuel Cell Store). The anodic compartment contained a carbon rod as CE and 0.01 M H_2_SO_4_ as anolyte. The cathodic compartment housed the WE and the RE, with acidified 0.1 M K_2_SO_4_ as the catholyte (pH 3). The GC method employed enabled gas detection every 11 min and the Faraday efficiencies (FE) toward all gaseous products were calculated according to following equation:

$$FE\;\left(\%\right)=\frac{I_{product}}{I_{total}}=\frac{10^{-6}\cdot {ppm}_{product}\cdot \frac{p\cdot \phi_{V}}{R\cdot T}\cdot z\cdot F}{I_{total}}\cdot 100$$

with ppm_product_ being the concentration of the product determined by GC (ppm), *p* being the system pressure (bar), ϕ_V_ being the volumetric gas flow rate (L/s), *R* being the ideal gas constant (L·bar/mol·K), *T* being the absolute temperature (K), *z* being the number of electrons transferred per mol of product, *F* being the Faraday constant (A·s/mol), and *I*_total_ being the total current (A).

The concentration of FA was quantified using a gas chromatograph (NEXIS GC-2030, Shimadzu) equipped with a mass spectrometer (QP 2020 NX, Shimadzu). Due to its inherent low volatility, FA was first derivatized to ethyl formate. To enhance the analytical signal and facilitate the transfer of the derivatized product into the gaseous phase, a Shimadzu HS-20 NX headspace sampler was utilized. Chromatographic separation of the analytes was achieved on a midpolarity cross-bond sylarylene phase column (SH-I-624Sil MS, 20 m, 0.18 mm ID, 1 μm film thickness). The FE toward FA was subsequently calculated according to following equation:

$$FE\;\left(\%\right)=\frac{m_{measured}}{m_{calculated}}=\frac{c_{product}\cdot V_{catholyte}}{\frac{I\cdot t}{F\cdot z}\cdot M_{product}}\cdot 100$$

with *c*_product_ being the concentration of the product (g/L), *V*_catholyte_ the volume of the catholyte solution (L), *I* the applied current (A), *t* the time (s), *F* the Faraday constant (A·s/mol), *z* the number of electrons transferred per mol of products, and *M* the molar mass of the product (g/mol).

## 3 Results and Discussion

### 3.1 Splitting of the Reduction Reactions

To determine the onset potentials for both the hydrogen evolution reaction (HER) and the CO_2_ reduction reaction (CO_2_RR), we performed CV measurements in an acidified 0.1 M K_2_SO_4_ (pH 3) catholyte solution purged with either Ar or CO_2_. As shown in Figure 1, the HER is split into two different regions: the diffusion-limited proton reduction region and the water reduction region. Specifically, the diffusion-limited proton reduction is driven by a limited bulk concentration of H_3_O^+^ (10^–3^ M) and is generally reported to occur at pH values ranging from 2 to 5.^30,31^ The onset for proton reduction consistently remains at −0.7 *V*_RHE_, regardless of whether the solution is Ar or CO_2_ purged. In contrast, the onset for water reduction exhibits a notable difference. This is attributed to the CO_2_RR occurring between the two hydrogen-forming reactions, which suppresses water reduction and, thus, shifts its onset. Figure 1 illustrates these three distinct reduction reactions in a CO_2_-saturated electrolyte (solid line), which are color-coded for clarity: blue represents the predominant formation of hydrogen, while green indicates the dominant formation of formic acid (FA). Clear and discernible changes in the CV are observed at approximately −1.1 and −1.4 *V*_RHE_, signifying that the CO_2_RR to FA achieves its highest selectivity within this particular potential range. The formation of any other product than H_2_ or FA can be excluded, based on GC screening of the gas and liquid phases, which is explained in the next section.

$$Proton\;reduction:,{2H}^{+}+2e^{-}\to H_{2}$$

$$CO_{2}\;reduction:,CO_{2}+2H^{+}+2e^{-}\to HCOOH$$

$$Water\;reduction:,2H_{2}O+2e^{-}\to H_{2}+2OH^{-}$$

**Figure 1:**
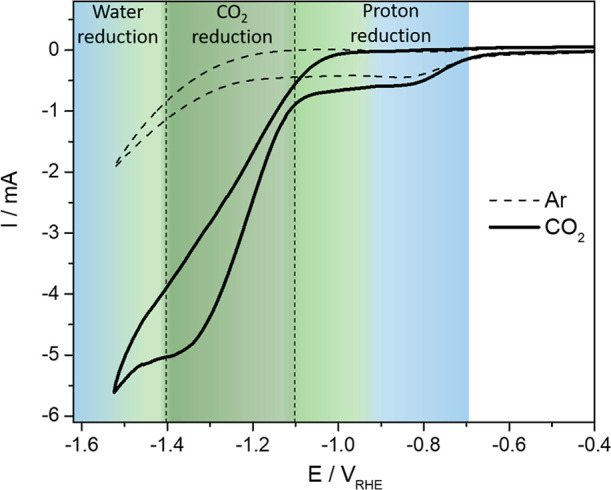
CVs of Bi/C in acidified 0.1 M K_2_SO_4_ (pH 3), purged with CO_2_ (solid line) and Ar (dashed line) as reference. Scan rate: 50 mV/s. In this mildly acidic electrolyte, the HER bifurcates into two distinct pathways: the diffusion-limited proton reduction and the water reduction. The CO_2_RR occurs in the potential region between the two HER pathways. The predominant regions for either hydrogen formation or CO_2_ reduction are highlighted in blue and green, respectively, with distinct changes in the CV at −1.1 *V*_RHE_ and −1.4 *V*_RHE_ (dashed lines), indicating a significant shift in selectivity.

### 3.2 Potential-Dependent Analysis of CO_2_ Reduction Reaction Products

To determine the nature and distribution of the products, potential-dependent GC studies were performed by applying potentials between −0.8 *V*_RHE_ and −1.5 *V*_RHE_, in 0.1 V steps. Each potential was held for 55 min, with continuous analysis of gaseous products every 11 min. In addition, aliquots were collected for liquid product analysis after the completion of each potential step. This methodology yielded five distinct measurements of the gas phase composition for each potential. The liquid phase composition was sampled once per potential, at the end of the potential step; this measurement therefore represents the accumulated product concentration over the entire duration of the applied potential. GC analysis (Figure 2) shows that formic acid and H_2_ are the sole products with a strong dependency of their respective FEs on the applied potentials. FA formation is already observed at −0.8 *V*_RHE_ with a FE of ∼30% (Figure 2), within the diffusion-limited proton reduction region. At −1.1 *V*_RHE_, a significant change in selectivity occurs: the CO_2_RR becomes the dominant reaction, with most of the available protons being utilized for FA formation instead of H_2_, which boosts its FE from 48.1% to 84.4%. This is visible in the sudden change in partial currents of H_2_ and FA (Figure 2), without any change in total current, and clearly indicates a shift in the reaction pathway from the formerly dominating proton reduction toward the production of FA. This observation emphasizes that in this potential region, proton reduction is suppressed by the CO_2_RR. Stepping further cathodically, the FE of FA formation increases to >90% along with an increase in the total cathodic current, which is exclusively used for the CO_2_RR, as evidenced by the partial current increase for FA. A second change in selectivity initiates at −1.4 *V*_RHE_ and leads to increasing H_2_ formation through the arising water reduction. At −1.5 *V*_RHE_, H_2_ formation becomes even more pronounced, which is accompanied by a continuous increase in both total cathodic current and H_2_ FE. This likely results from an increase in surface pH caused by OH^–^ formation near the electrode,^38^ which might not only affect the reaction dynamics but the surface chemistry as well.

**Figure 2:**
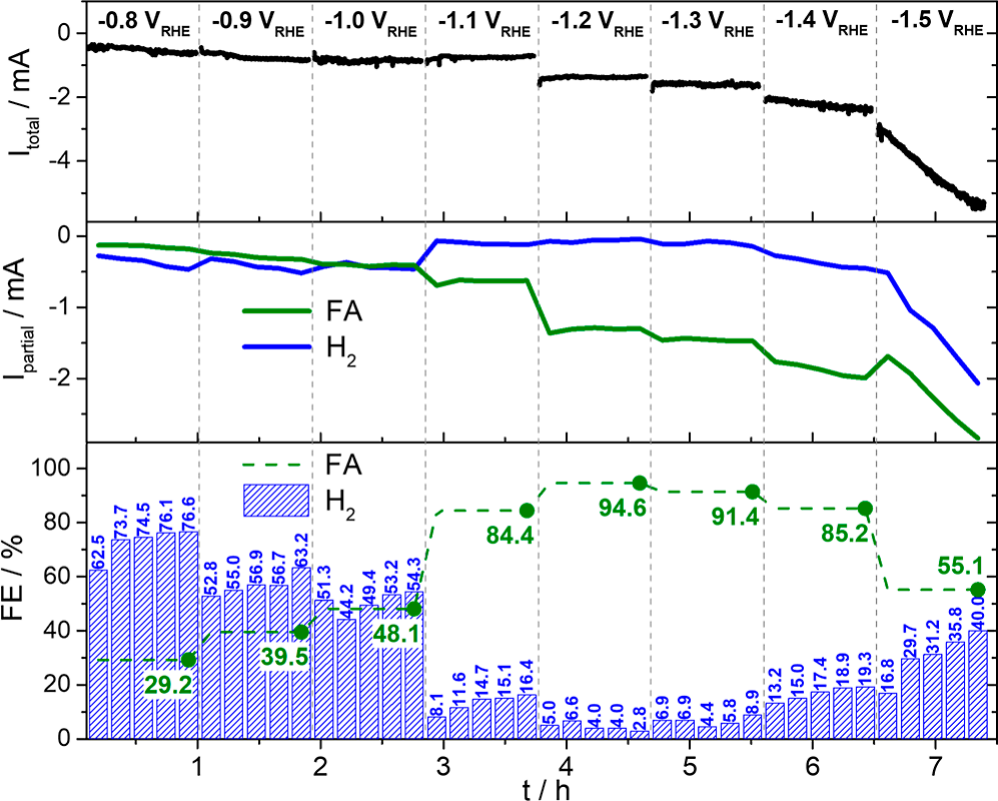
Potential-dependent online GC-measurement of Bi/C in CO_2_-saturated acidified 0.1 M K_2_SO_4_ (pH 3). The figure presents the total currents (black line), the partial currents for FA (green line) and H_2_ (blue line), and the FEs for FA (green dashed line) and H_2_ (blue bars) formation for each investigated potential. There are two distinct selectivity changes visible: (i) proton to CO_2_ reduction at −1.1 *V*_RHE_ and (ii) CO_2_ to water reduction at around −1.4 *V*_RHE_. The first selectivity shift involves the suppression of proton reduction by the CO_2_RR, evidenced by a drop in H_2_ partial current with no change in total current. The second selectivity shift is marked by an increase in total cathodic current and H_2_ FE, which likely arises from a localized pH increase at the catalyst surface due to OH^–^ formation via water reduction.

These findings highlight that at pH 3, the proton reduction does not effectively compete with the CO_2_RR, as the reduction of CO_2_ to formic acid effectively suppresses it. However, water reduction demands careful attention during scale-up, given its drastic impact on both the total current and the FE toward H_2_ over time.

### 3.3 The Important Role of Surface Oxides

To investigate the Bi/C catalyst’s long-term stability, a constant potential of −1.3 *V*_RHE_ was selected, where the partial current for FA formation is maximal, without any significant change in the H_2_ partial current (see Figure 2). In analogy to the GC study presented in Figure 2, the gaseous phase was analyzed every 11 min, while a liquid sample was taken every 2 h. The FE toward FA formation consequently represents the average selectivity over that 2 h period. The expected trend is inversely correlated to the FE of H_2_ formation due to the exclusive formation of these two products. The catalyst initially demonstrates excellent selectivity toward FA, surpassing an averaged FE of around 97% during the first 2 h (see Figure 3). Nevertheless, the FE value decreases to about 70% after 6 h.

**Figure 3:**
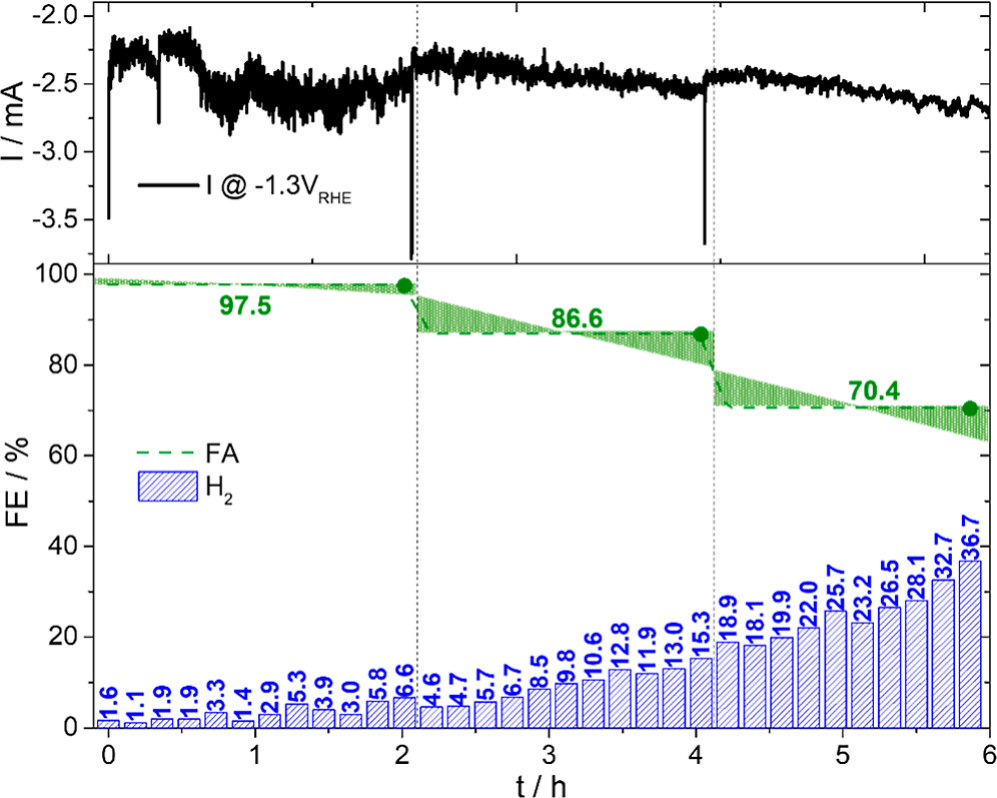
Online GC measurement at −1.3 *V*_RHE_ with Bi/C in CO_2_-saturated acidified 0.1 M K_2_SO_4_ (pH 3). The figure shows the total current (black line) and the FE toward H_2_ (blue bars) and FA (green dashed line) formation. The initial 2 h show a remarkably high average FE of FA formation of >95%. The FA FE declines to <70% within 6 h. The reason for this drastic decrease is attributed to a change in local pH and to the reduction of the inherently formed surface oxide.

To better understand this continuous shift in selectivity and the corresponding deactivation of the catalyst, XPS (Figure 4a) and CV (Figure S2) studies were conducted. They reveal a clear correlation between the oxidation state of Bi at the surface and its electrocatalytic response. XP spectrum (Figure 4a) of the pristine catalyst surface prove that, prior to each electrochemical measurement, the surface is oxidized, evidenced by a spin–orbit couple observed at 159.5 and 164.5 eV. This oxidation likely occurs at the Bi nanoparticle surfaces during ink formation in ambient air, while the bulk Bi nanoparticle nature remains metallic (Figure S1a). The corresponding CV (Figure S2a) of the surface-oxidized catalyst consequently exhibits three distinct reduction reaction regions: proton, CO_2_, and water reduction. After applying a reductive potential of −1.1 *V*_RHE_ for 3 h, the XP spectrum (Figure 4a) shows a significant change in the surface chemistry, revealing the presence of both Bi suboxides/hydroxides (158.8/164.0 eV) and metallic Bi (156.5/162.0 eV). The electrochemical response (Figure S2b) shows a concomitant change: upon surface reduction, the three distinct reduction regions merge into two regions through an onset shift of the water reduction to less negative potentials and a slow disappearance of the CO_2_ reduction range. This change is likely attributed to a decreased overpotential for water reduction on the partially reduced surface. The observed decrease in FE toward FA formation can therefore be attributed to the reduction of the surface oxide to metallic Bi. The reduced species exhibits a lower overpotential for water reduction, which leads to a competition between the water and the CO_2_ reduction processes at −1.3 *V*_RHE_. This competition and the concomitantly induced local pH shift due to the water reduction is the reason for the observed drop in FE toward FA formation. Consequently, the Bi_2_O_3_ species provides a broader potential window for the CO_2_RR before competition from the water reduction reaction is initiated, relative to the reduced Bi species.

**Figure 4:**
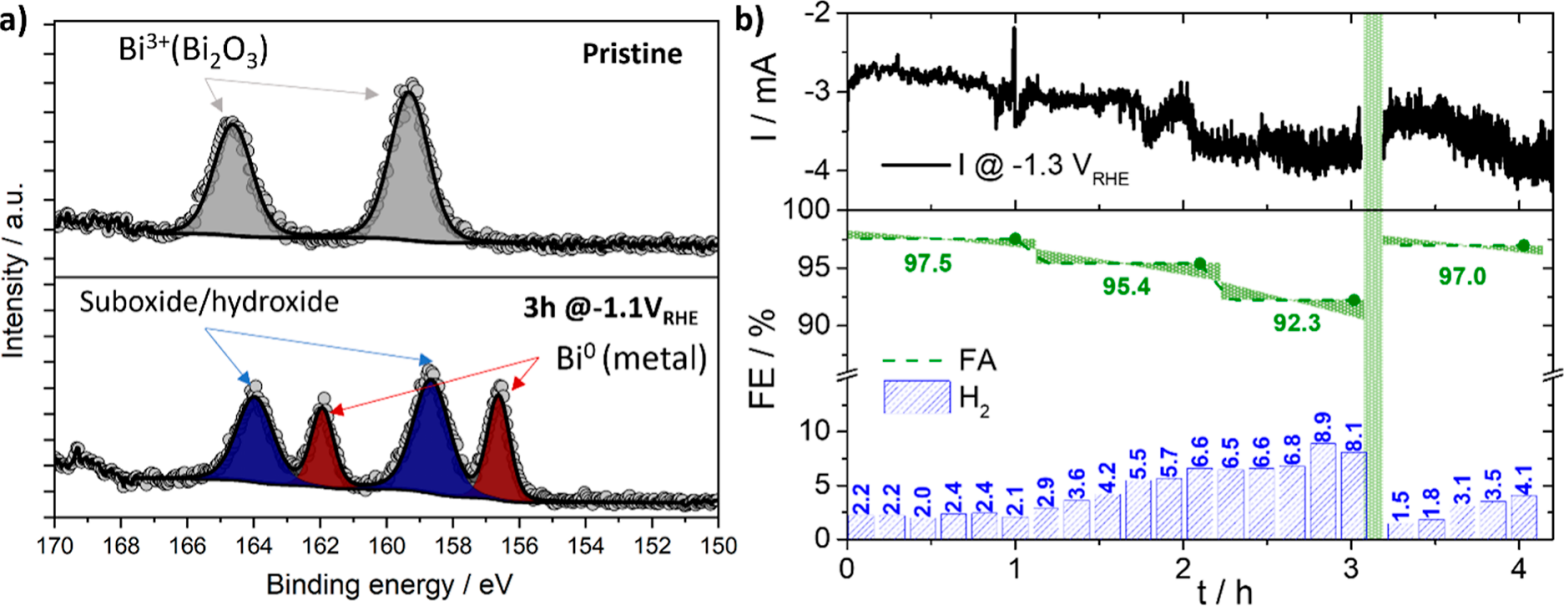
(a) XPS measurements (Bi 4f region) of Bi/C detailing that the initial surface composition consists of pure surface Bi_2_O_3_. Applying a reductive potential of −1.1 *V*_RHE_ for 3 h reduces the surface oxide into metallic bismuth and various suboxides or hydroxides. (b) Online GC measurement at −1.3 *V*_RHE_ with Bi/C in CO_2_-saturated acidified 0.1 M K_2_SO_4_ (pH 3). This graph displays the total current (black line) and the FE to H_2_ (blue bars) and to FA (green dashed line). After 3 h of constant potential application, an oxidation cycle to 0.8 *V*_RHE_, highlighted in green, was performed, and subsequently, the same potential was reapplied. The observed decrease in FE for H_2_ demonstrates that a single oxidation cycle can effectively restore the catalysts’ initial selectivity.

Intriguingly, the initial electrocatalytic behavior is recovered by a single oxidation cycle (Figure S2c,d). This behavior is demonstrated in Figure 4b, where a potential of −1.3 *V*_RHE_ was applied for 3 h, followed by a single oxidation cycle (Figure S2c), after which the same reductive potential of −1.3 *V*_RHE_ was reapplied. This unambiguously led to a decrease of the FE toward H_2_ and an increase of the FE toward FA formation, which demonstrates that the desired selectivity can be fully restored by a single oxidation cycle. It is noteworthy that a potential step to an anodic potential of 0.8 *V*_RHE_ was also tested as a methodology to reoxidize the catalyst surface. This approach resulted, however, in diminished catalyst stability, which was the reason for the application of an oxidation cycle. Specifically, after the potentiostatic measurement, the potential was cycled from the applied reductive potential up to 0.8 *V*_RHE_ and back with a scan rate of 50 mV/s. Further optimization of the exact procedure for this oxidative reactivation of the catalyst has yet to be undertaken but is beyond the scope of this report.

Due to the identified crucial role of the surface oxide for FA selectivity, supplementary experiments employing commercially available Bi_2_O_3_ nanoparticles were conducted. The overall electrochemical response of this reference material is determined through recording a CV in CO_2_-saturated 0.1 M K_2_SO_4_ (pH 3) (Figure S3). It shows an electrocatalytic fingerprint identical with that of the Bi/C catalyst. This confirms that the oxide is responsible for the catalytic selectivity toward FA formation (see Supporting Note 1 for a detailed description). Long-term measurements with Bi_2_O_3_ (Figure S4) exhibit a slightly lower initial FA FE of ∼90% and a comparatively slower FE decrease to ∼85% after 6 h. XPS studies (Figure S5) reveal that, similar to the Bi/C catalyst, the pristine Bi_2_O_3_ catalyst undergoes reduction to metallic Bi over time at −1.3 *V*_RHE_. The lower initial selectivity of the Bi_2_O_3_ reference material toward FA formation is attributable to a core/shell morphology of the Bi/C catalyst, where a Bi core and a Bi_2_O_3_ shell could offer catalytic benefits for the CO_2_RR due to a higher electronic conductivity of the Bi core, while the Bi_2_O_3_ shell simultaneously enhances the catalytic selectivity. This idea, however, needs further investigation to determine whether such a core/shell structure is indeed responsible for the enhanced performance.

This study demonstrates that despite a loss of selectivity over time, a single oxidation cycle largely restores the initial performance of the Bi/C catalyst. However, further research is crucial to achieving selectivities exceeding 95% in acidic media over extended periods. Our findings indicate that several strategies are promising for enhancing practical performance: optimizing the operational potential window to mitigate the impact of water reduction, sustainably stabilizing the thin surface oxide shell during the CO_2_RR, and exploring continuous regeneration of the oxide surface using pulsed electrolysis. All of these approaches have their distinct advantages and disadvantages and require dedicated further investigation.

## 4 Conclusions

In this study, we employed electrochemistry coupled with analytical techniques to investigate the selectivity of the nanoporous Bi/C catalyst for the CO_2_RR for FA under mildly acidic conditions. We find that FA formation manifests precisely between the onsets of the two HER pathways, i.e., the diffusion-limited proton reduction at lower overpotentials and the water reduction at higher overpotentials. The CO_2_RR effectively suppresses proton reduction due to the preferential consumption of protons for FA formation. This enables the Bi/C electrocatalyst to achieve high FE exceeding 95% for FA production at potentials of −1.2 to −1.3 *V*_RHE_.

Long-term potentiostatic GC studies conducted at −1.3 *V*_RHE_ reveal a time-dependent decrease in FE. This occurs due to the reductive transformation of the catalyst surface from Bi_2_O_3_ to metallic Bi. Ambient air exposure during ink preparation inherently leads to the formation of the surface oxide Bi_2_O_3_ on Bi/C, which we identified to be responsible for the high catalytic selectivity toward FA formation, while the metallic Bi core guarantees high electronic conductivity. The observed decrease of this FE from >95% to <70% is intimately linked to a change in surface chemistry, i.e., the partial reduction of the surface oxide. The initial high selectivity can be restored through one oxidation cycle.

Analysis of the initial FE values for Bi/C and the Bi_2_O_3_ reference suggests a synergistic effect that originates from the intrinsically formed Bi core/Bi_2_O_3_ shell structure. This synergy likely arises from the enhanced electronic conductivity of the metallic Bi core coupled with the increased catalytic activity and exceptional selectivity toward FA formation of the Bi_2_O_3_ shell.

## Supplementary Material



## Data Availability

The data that support the findings of this study are openly available in InvenioRDM at 10.48323/t7gm2-9fa89.

## Notes

TEM and XRD measurements of the pristine Bi nanoparticles, CV measurements after different applied potentials, reference CV measurements of Bi_2_O_3_ nanoparticles, and long-term study of the Bi_2_O_3_ catalyst (PDF)
