# Supplementary material for: Boosting Formic Acid Production in Mildly Acidic Media—The Role of Native Surface Oxide on the CO2 Reduction Performance
Source: ACS Omega. 2025 Nov 11;10(46):56863–9. doi: 10.1021/acsomega.5c10136 (PMC12658832; doi:10.1021/acsomega.5c10136)
Supplement: Supplementary file 1 [file ao5c10136_si_001.pdf]

Supplementary Information

Boosting formic acid production in mildly acidic media - The role of native surface oxide on the CO_2 reduction performance

Thomas Mairegger,[†1,2] *Christoph Griesser,*[1] *Sergio Diaz Coello,*[1] *Michael Höltig,*[3] *Christoph Gimmler,*[3] *Philipp Stadler,*[2] *Alexander Beck,*[2] *and Julia Kunze-Liebhäuser*[*1]

[1]Department of Physical Chemistry, University of Innsbruck, Innrain 52c, 6020-Innsbruck, Austria.
[2]Net Zero Emission Labs GmbH, Sinning 1, 83101-Rohrdorf, Germany
[3]Fraunhofer IAP-CAN, Grindelallee 117, 20146-Hamburg, Germany

*Corresponding author: Julia.Kunze@uibk.ac.at

This PDF file includes:

Supplementary figures
Supplementary notes

Supplementary Figure 1: X-ray diffraction (XRD) and transmission electron microscopy (TEM)

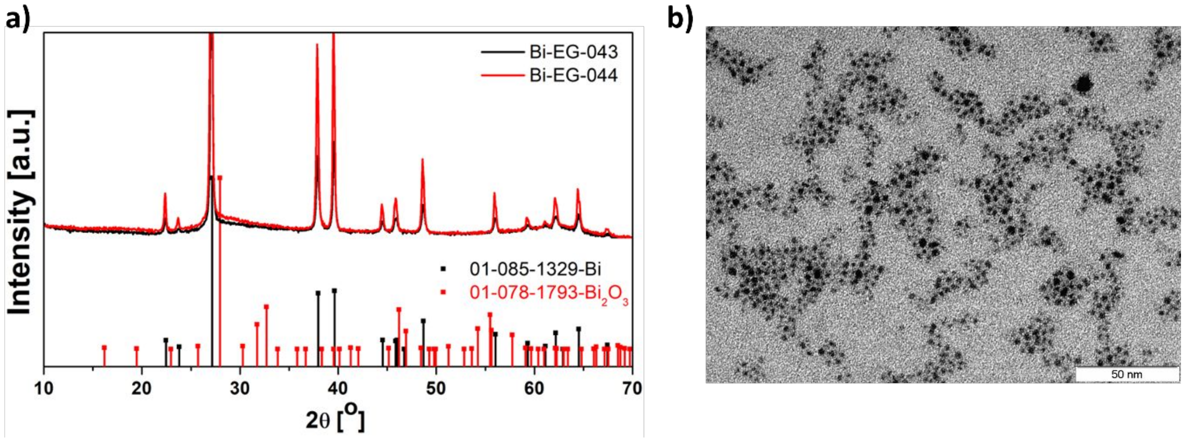

Figure S1: a) XRD patterns of synthesized Bi nanoparticles from two distinct batches (red and black). The diffractograms confirm that the nanoparticles are pure Bi, clearly distinguishing them from Bi_2O_3. The peak positions for Bi (black) and Bi_2O_3 (red) from the database ICDD (International centre for diffraction data) are shown below the diffractograms. b) TEM image of the synthesized Bi nanoparticles, showing average particle sizes of 3.1 ± 0.8 nm.

Supplementary Figure 2. Electrochemical response of different oxidation states of Bi

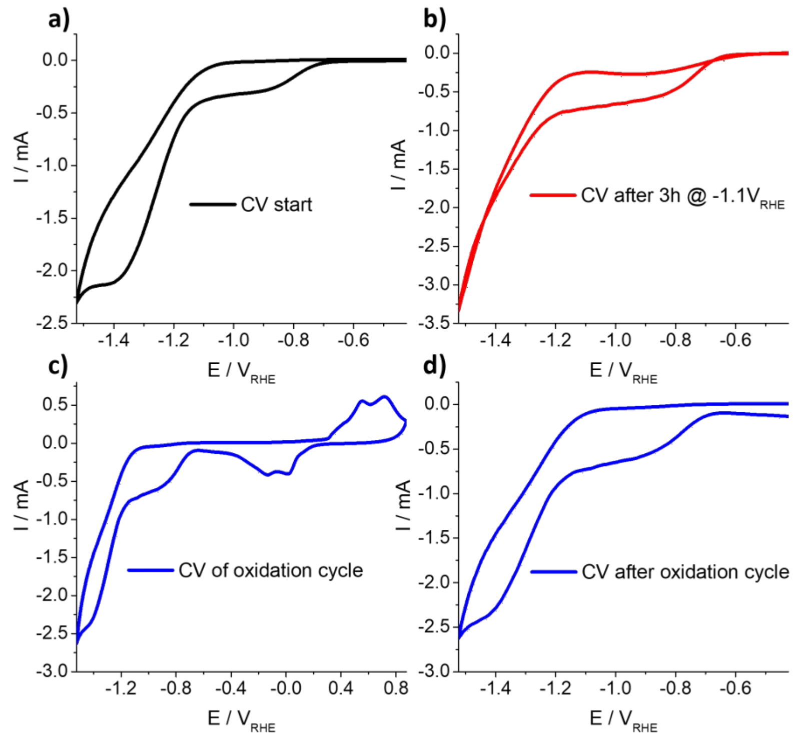

Figure S2: CVs of Bi/C in CO_2 saturated acidified 0.1 M K_2SO_4 (pH 3) with a scan rate of 50 mV/s. XP spectra (Figure 4a) reveal that the initial surface is oxidized; after applying a reductive potential of -1.1 V_{RHE} for three hours the surface undergoes reduction. This change in oxidation state can be correlated to a change in the electrocatalytic response. a) The oxidized surface shows three distinct reductions reactions (see main text) and b) the partially reduced surface causes the water reduction to merge with the CO_2 reduction, likely due to a decrease in its overpotential. c)-d) The initial electrochemical behavior, is recovered by a single oxidation cycle.

Supplementary Note 1. Reference CV measurement of Bi_2O_3 nanoparticles

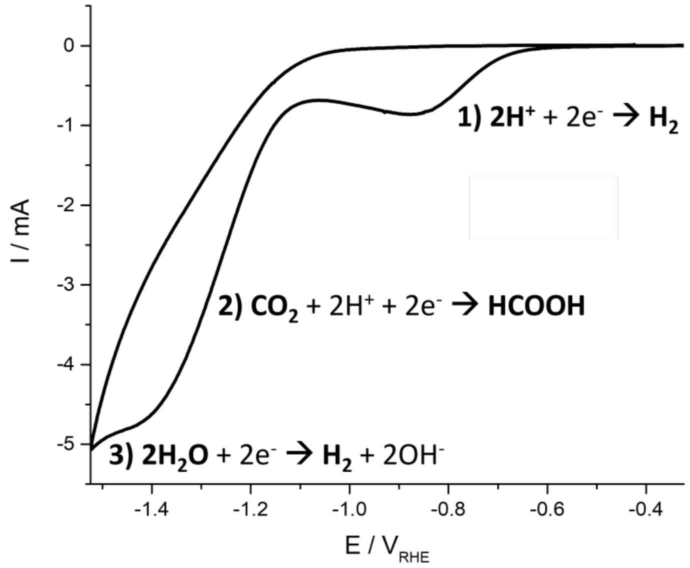

Figure S3: CV of Bi_2O_3 in CO_2 saturated acidified 0.1 M K_2SO_4 (pH 3). The CV exhibits the three expected reduction reactions: proton reduction, CO_2 reduction, and water reduction. This consistent electrochemical signature confirms that the surface oxide (Bi_2O_3) is indeed the catalytically active species driving these reactions and enabling a FE towards FA formation of >90%.

To investigate the electrochemical behavior of Bi_2O_3 nanoparticles in CO_2 saturated acidified 0.1 M K_2SO_4 (pH 3), we performed a CV measurement (Figure S3). The CV shows an identical electrocatalytic response as that recorded with Bi/C in the same electrolyte (Figure 1). This identical fingerprint strongly emphasizes that the Bi_2O_3 is the catalytically active species for the CO_2 reduction to FA. It confirms the existence of the three distinct reduction reactions (see main paper) that can therefore be considered the characteristic fingerprint of Bi oxide and Bi oxide covered Bi catalysts in 0.1 M K_2SO_4 at pH 3.

Supplementary Note 2. Long-term studies of the Bi_2O_3 catalyst

Figure 4a revealed that the surface oxide of Bi is likely the active species in the selective reduction of CO_2 to FA. Therefore, analogous long-term potentiostatic GC analysis employing Bi_2O_3 nanoparticles were conducted at the same potential as for the Bi/C catalyst. Gaseous products were analyzed at 11-minute intervals, while liquid samples were collected every three hours, with the FE of FA formation representing the average selectivity observed over each period. Comparison of the Bi_2O_3 nanoparticles with the Bi/C catalyst reveals a lower initial FE towards FA of ~90%, which decreases slower than that of Bi/C to ~85% after six hours.

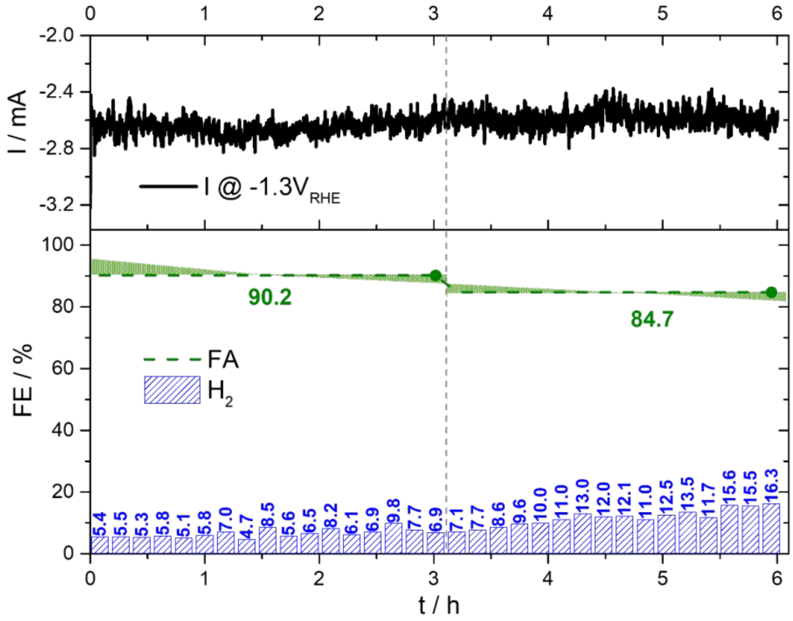

Figure S4: Online GC study at -1.3V_{RHE} of Bi_2O_3 in CO_2 saturated acidified 0.1 M K_2SO_4 (pH 3). The figure shows the total current (black line) and the FE towards H_2 (blue bars) and FA (green dashed line).

The higher initial selectivity observed with the Bi/C catalyst (Figure 3) is attributable to a core/shell morphology, where a Bi core and a Bi_2O_3 shell could offer catalytic benefits for the CO_2RR due to a higher electronic conductivity. The observed FE decrease for FA formation indicates that the pure Bi_2O_3 catalyst also reduces to metallic Bi over time. Monitoring the chemistry evolution with XPS indeed reveals that the initial oxide surface predominantly consists of Bi_2O_3 (spin orbit couple at 159.5 eV and 165.0 eV) and is reduced in part to metallic Bi (156.5/162.0 eV) after five hours at a potential of -1.3 V_{RHE} (see Figure S5).

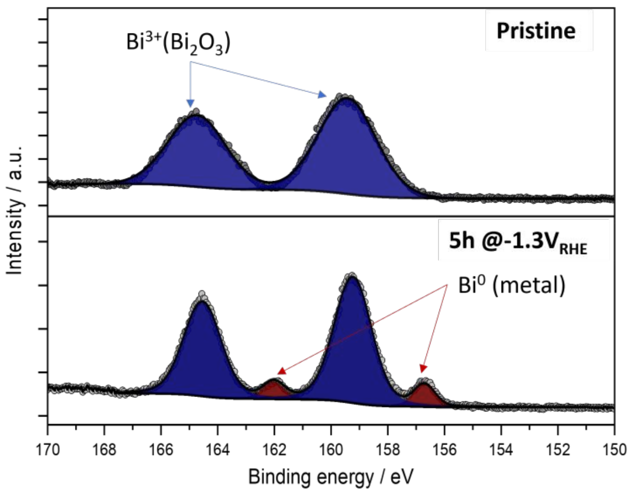

Figure S5: XP spectra (Bi 4f region) of the Bi_2O_3 nanoparticles confirm that its initial surface composition is predominantly Bi_2O_3. After application of a reductive potential of -1.3V_{RHE} metallic Bi is detected.
